# Quantification of Pharmaceuticals in Sludge Produced from Wastewater Treatment Plants in Jordan and Environmental Risk Assessment

**DOI:** 10.3390/toxics14010062

**Published:** 2026-01-08

**Authors:** Othman Almashaqbeh, Christina Emmanouil, Layal Alsalhi

**Affiliations:** 1 Emerging Pollutants Research Unit, Royal Scientific Society, P.O. Box 1438, Amman 11941, Jordan; othman.mashaqbeh@rss.jo (O.A.); leial.salhi@rss.jo (L.A.); 2 School of Spatial Planning and Development, Aristotle University of Thessaloniki, 54124 Thessaloniki, Greece

**Keywords:** sludge, organic amendments, antibiotics, risk assessment, terrestrial toxicity, circular economy, Jordan

## Abstract

Sewage sludge is increasingly recognized as a major reservoir for pharmaceuticals and emerging contaminants that are only partially removed by conventional wastewater treatment. This study provides the first comprehensive assessment of these contaminants in biosolids generated from ten major wastewater treatment plants (WWTPs) across Jordan. Different pharmaceuticals were quantified in the sludge samples generated. The results revealed concentrations ranging from 10 to over 2000 µg kg^−1^, with antibiotics typically showing the highest enrichment (e.g., ciprofloxacin up to 2165 µg kg^−1^, ofloxacin up to 303 µg kg^−1^). Anti-inflammatory compounds such as diclofenac reached 196 µg kg^−1^, while the antimicrobial triclosan exceeded 4700 µg kg^−1^ in some sludge samples. Carbamazepine, a recalcitrant antiepileptic drug, ranged between 50 and 223 µg kg^−1^, reflecting both widespread use and strong persistence. Elevated levels of quaternary ammonium compounds (QACs) were also detected. The highest levels were generally associated with large urban WWTPs and plants receiving industrial discharges. Environmental risk assessment (ERA) indicated that the risk for soil biota was acceptable for most cases for low application doses (5–10 t/ha) except for WWTP6-MD, WWTP8-S, and WWTP9-IC, where the risk was non-acceptable. Severe limitations in the risk assessment were noted: reliable toxicity endpoints in terrestrial soil organisms such as microbiota, collembola, and earthworms are few, while deriving endpoints via aquatic available data is not always reliable. Overall, the findings demonstrate that Jordanian sewage sludge contains environmentally relevant levels of pharmaceuticals and QACs and that risk assessment is, therefore, pertinent before any stabilization and realistic land application scenarios are chosen.

## 1. Introduction

Urban wastewater consists of liquid waste generated from everyday household activities (such as sanitation, cooking, laundry, dishwashing, etc.), offices, institutions, commercial facilities, and small industries. Its main components are water, pathogens, and mixtures of inorganic and organic pollutants, which render it unsuitable for use and potentially harmful to aquatic organisms in receiving waters. Collected urban wastewater is treated in Wastewater Treatment Plants (WWTPs), critical technical infrastructures for any city. The vast majority of WWTPs employ biological treatment processes—specifically the activated sludge method—where the sludge is dispersed in tanks or attached to a solid substrate through which wastewater flows. The activated sludge process is essentially synonymous with the term “secondary treatment” [1,2].

Wastewater treatment plants (WWTPs) are increasingly receiving wastewater containing complex mixtures of emerging contaminants, including pharmaceuticals and personal care products (PPCPs), disinfectants, endocrine-active compounds, and a variety of industrial chemicals [3,4]. Emerging contaminants may be partially removed by secondary treatment and through other advanced treatment processes [5,6,7]. However, it is also possible that pharmaceuticals and their metabolites may accumulate in sewage sludge, the excess sludge produced by the increased biomass of the activated sludge, due to their high persistence, strong sorption affinity, or limited biodegradability. Consequently, sludge from WWTPs has become a major environmental reservoir for emerging organic contaminants [8], and vast research is performed on its contamination with antibiotics, analgesics, anti-inflammatory drugs, antiepileptics, antimicrobials, and quaternary ammonium compounds (QACs) [9,10,11,12].

Non-stabilized sludge is potentially hazardous due to its pathogenic content; therefore, sludge must undergo a series of treatments to ensure its safe removal from the WWTP and possible reuse, either within or outside the facility [2,13]. Properly treated sludge is referred to as biosolids (BS) [14]. This is not considered waste anymore; it is a substrate full of nutrients that can potentially aid plant development and augment crop yield [15]. The BS application is particularly suitable for poor agricultural lands, helping restore soil organic content. Increasing organic matter boosts crop productivity, and it can also stimulate microbial populations and soil biological activity [16].

Furthermore, BS contain significant quantities of nitrogen and phosphorus derived from nitrification–denitrification processes. These nutrients can support plant growth and reduce or eliminate the need for synthetic fertilizers.

It is, therefore, evident that BS land spreading is a double-edged sword, possessing both advantages and disadvantages [17]. Recognizing this risk of pollutant recycling within the environment, the USA has established regulatory requirements under the Part 503 Rule, addressing the impacts of BS land application [18]. Within the European Union, Directive 86/278/EEC on the “use of sewage sludge in agriculture” (adopted in 1986) prescribes maximum allowable metal concentrations in sludge intended for agricultural use, along with maximum annual loadings of those metals that may be applied to soils. Since then, several Member States have adopted national laws with stricter or additional limits than those in the Directive. These limits may be pertinent to metals but they may also refer to other persistent pollutants such as Halogenated Aromatic Compounds (HACs) due to their persistence, bioaccumulation, and toxicity. The original Directive made no mention of HACs [19] A future revision of the Directive at the EU level is expected [20] and HACs are of particular concern [21].

Regarding Jordan, national legislative limits for sludge use make an important distinction between uses; there is a distinction between soil improvement (organic amendment) and application or disposal in landfills, as well as a categorization of sludges (Class I to III) (Jordanian Standard JS 1145:2016) [22]. Therefore, the landfilling option is still available in Jordan, while essentially in the EU it is now extinct [23]. Landfilling can help dispose of problematic and highly polluted batches; however, it does not exploit the high resource potential of this biodegradable waste [24]. It would be ideal to minimize final disposal options while utilizing waste as a substrate for new and important exports [25,26].

To summarize, the application of sludge in the form of BS as fertilizer on agricultural land is an appealing solution because of its agronomic benefits [27,28]. However, this use must comply with strict specifications to safeguard the environment and public health, since toxic contaminants—such as metals, dioxins, organochlorine derivatives, emerging pollutants, and pathogenic microorganisms—may be released from partially treated BS under certain conditions [29]. Hence, quantification of priority pollutants in BS before land application is imperative [30]. However, the actual effect of pollution on ecosystems is quantified through the method of risk assessment, which is defined according to USEPA as “the process for evaluating how likely it is that the environment might be impacted as a result of exposure to one or more environmental stressors, such as chemicals, land-use change, disease, and invasive species”. Risk assessment has been a valuable tool for scientists and policy makers, enabling them to identify, quantify, and manage risks to safeguard human health and the environment.

In this context, the present research quantifies numerous emerging pollutants (antibiotics, analgesics, other pharmaceuticals, QACs, and other antibacterial drugs) commonly found in sludge, and it assesses their ecotoxicological potential for soil species, for several WWTPs in Jordan. This is the first report on emerging contaminants in sludge in Jordan, which also includes different treatment processes of the sludge produced. This research can also enrich the ever-expanding database on biosolids hazard and risk characterization to enable more sustainable use patterns of biodegradable waste.

## 2. Materials and Methods

### 2.1. WWTPs Characteristics and Sampling Procedure

#### Collection of Sludge Samples

**Figure 1:**
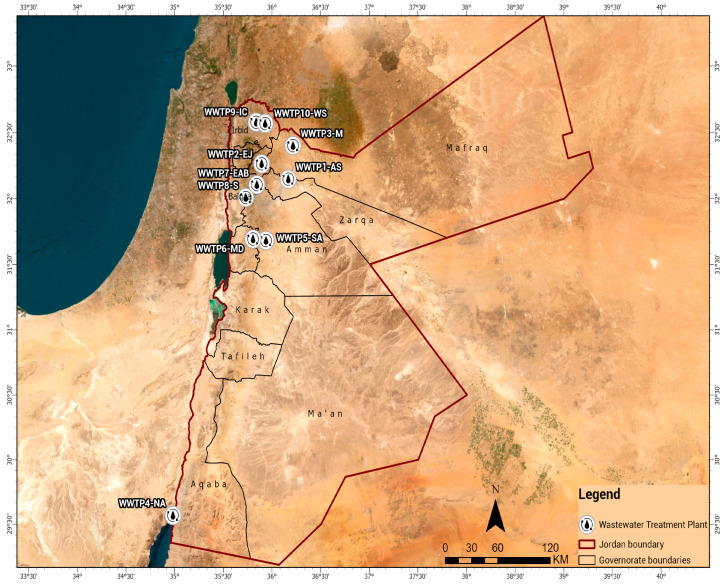
Geographical distribution of the ten WWTPs.

Sludge samples were collected from ten municipal wastewater treatment plants (WWTPs) distributed across different climatic and geographical regions of Jordan, as shown in Figure 1. The selected WWTPs represent a wide range of treatment capacities and operational technologies, including biological activated sludge systems, drying beds, and thickener-based dewatering units. Each facility serves a distinct population and hydrological basin, providing a representative overview of sludge characteristics across the country. Some characteristics of the WWTPs are shown in Table 1, and a map of all examined WWTPs is shown in Figure 1.

Sampling was conducted in April 2022, a period characterized by moderate climatic conditions and stable operational performance of the WWTPs. A single composite sludge sample was collected from each plant at the final sludge treatment stage, including dewatering outlets, drying beds, thickeners, or filter press outlets (Table 2). These points were selected to represent the final stabilized sludge typically destined for reuse or disposal.

Sludge annual production was derived from data from Table 1, applying the following formula

(1) $$Sludge\;production\;C\left(\frac{t}{y}\right)=Flow\left(\frac{m^{3}}{y}\right)\cdot TSS\left(\frac{mg}{L}\right)\cdot 10^{-6}$$

with

Flow: the influent rate per year
TSS: total suspended solids in the inflow
10^−6^ conversion factor (L to m^3^, mg to t)

At each sampling location, sludge (approximately 1 kg) was collected, taking all necessary precautions (gloves, mask) with pre-cleaned stainless-steel scoops into acid-washed high-density polyethylene (HDPE) containers. Samples were immediately sealed, labeled, and stored at 4 ± 1 °C in insulated ice boxes during transport to the laboratory.

### 2.2. Sample Extraction

The collected sludge samples were freeze-dried for 24 h (DC401, Yamato, Tokyo, Japan) before the extraction of pharmaceuticals following the method specified by [31]. To make the samples homogeneous, all dried sludge samples were mixed using a blender (Geepas, Ningbo, China). In brief, dried sludge samples (1.0 g) were extracted with methanol (20 mL) and formic acid (0.1 mL) under ultrasonic treatment (VWR, USC-THD, Radolfzell, Germany) for 30 min at 10 °C. All extracted samples were centrifuged at 3000 rpm for 20 min (Centurion K2015R Refrigerated Centrifuge, Chichester, UK). The supernatant was decanted into 20 mL glass test tubes, and the residue was extracted once more using fresh solvent. The decanted supernatant was evaporated (Biotage, Turbo-Vap LV, South Wales, UK) until 1 mL final volume. Deionized water (20 mL) was added to the evaporated samples. The polymeric Hydrophilic–lipophilic balance (HLB) Oasis 6cc cartridge (Oasis, Milford, MA, USA) was conditioned by passing 6 mL of acetone and 6 mL of methanol sequentially, followed by 6 mL of distilled deionized water. Then, the prepared sample passed through the solid phase extraction (SPE) cartridge at a flow rate of 10 mL/min or less. After the end of the extraction, the cartridge was rinsed with 5 mL of deionized water, and room air was allowed to flow through the cartridge by continued suction for at least 5 min to dry the cartridge. All extracted samples were eluted by adding methanol (10 mL). The supernatant was collected in a glass test tube and evaporated using Turbo-Vap (Biotage, South Wales, UK) to a 5 mL final volume.

For quantification of quaternary ammonium compounds, each sample was weighed exactly (50 ± 1 mg). The solid material was extracted twice using a mixture of acetonitrile and aqueous hydrochloric acid at 50 °C in a sonication bath. Each time, the supernatant was collected after centrifugation at 4500 rpm and underwent a dispersive SPE with primary-secondary-amine (PSA) as a clean-up step, followed by a phase separation with anhydrous MgSO_4_. The solvent was removed by applying a nitrogen stream at 40 °C, and the sample was reconstituted in 80% *v*/*v* aqueous methanol.

### 2.3. Chemical Analysis

Pharmaceutical compounds were quantified using a SCIEX Triple Quad 5500 LC–MS/MS system (AB SCIEX Triple Quad 5500, Framingham, MA, USA) equipped with an electrospray ionization (ESI) source operating in both positive and negative ion modes, depending on the analyte. Chromatographic separation was achieved on a C18 column (2.1 × 100 mm × 30 mm, 2.7 μm particle size) maintained at 30 °C. The mobile phase consisted of Solvent A: water with 0.1% formic acid and Solvent B: acetonitrile with 0.1% formic acid, delivered under a 13 min gradient program at a flow rate of 0.4 mL min^−1^. The injection volume was 10 μL.

Mass spectrometric parameters were optimized as follows: ion spray voltage 5500 V, curtain gas 60, gas 1 and gas 2 at 60 psi, and ion source temperature 550 °C. Instrumental detection capability of the LC–MS/MS system was 1 ppb under optimized operating conditions. Compound-specific LOQ values were determined separately for water and soil matrices as shown in Supplementary Materials. Method performance and analytical accuracy were assessed using external solvent-based calibration curves, demonstrating satisfactory linearity for all analytes, in the absence of matrix-matched calibration for sludge samples.

Quantification of quaternary ammonium compounds was conducted with a QTrap 4500 Triple-Quadrupole MS/MS detector from SCIEX (Framingham, MA, USA) equipped with a Shimadzu HPLC system (Shimadzu Corp, Dubai, United Arab Emirates).

### 2.4. Risk Assessment Calculations

A probabilistic risk assessment was performed for most of the substances that consistently gave concentrations above the limit of quantitation according to the formula below:

(2) $$RQ=\frac{PEC_{soil}}{PNEC_{soil}}$$

with RQ: risk quotient. PEC: Predicted Environmental Concentration in soil. PNEC: Predicted. No Effect Environmental Concentration for soil biota

PEC soil is calculated specifically for BS land spreading according to the following formula [32].

(3) $$PEC_{soil}=\frac{C_{sludge}\cdot AR\cdot (1-DF)}{BD\cdot DEPTH}$$

with C_sludge_: concentration measured in the biosolid (mg/kg dw). AR: application rate in kg dw/m^2^. DF: degradation fraction. BD: soil bulk density in t/m^3^. DEPTH: depth of incorporation in soil in m

AR was calculated as a series of possible scenarios of 5, 10, and 20 t/ha, values frequently examined in ecotoxicity experiments on biosolids [13].

BD was equal to 1.3 t/m^3^ and DEPTH equal to 20 cm [32]. Since no data on degradation was available, a worst-case scenario of DF = 0 was assumed.

However, in order to refine the assessment, a time-weighted PECsoil was calculated for the substances carbamazepine, ciprofloxacin, diclofenac, lincomycin, ofloxacin, and triclosan according to the following formulas [32].

(4) $$PEC_{soil\_twa}=PEC_{soil}\cdot (1-e^{-kt})/kt$$

(5) k=ln2/DT50

with k: first-order degradation rate constant. DT50: dissipation time in days. t: time duration in days equal to 30 days (approximately a short-term bioassay).

DT50 values that ideally correspond to field data of soil amended with BS were used, and they are shown in Table 2.

**Table 2:** DT50 values used for PECsoil_TWA derivation and related references.

Substance	DT50 Value in Soil (d)	References
Carbamazepine	97.6	[33]
Ciprofloxacin	5 years	[34]
Diclofenac	8.56	[35]
Lincomycin	19.4	[36]
Triclosan	-	[37]

Carbamazepine DT50 was quoted from [33] from a field study, which, however, was less conservative than lab-based studies [38]. Ciprofloxacin DT50 was quoted from [34] based on an extensive search on ciprofloxacin dissipation in BS amended soils and it was assumed as “conservative”. Diclofenac DT50 was derived from a lab-based study on BS amended soil, and it was equal to 8.56 d [35]. This is considered appropriate since similar values were found for agricultural soils spiked with diclofenac [39]. Lincomycin DT50 was calculated from a field experiment (the worst of both values) with manure applied data [36]. In a 366-day field experiment in South Australia, no significant dissipation of triclosan was noted in BS-amended soils [37], so here triclosan was also assumed stable.

Regarding PNECs, terrestrial toxicity databases are notoriously less populated than aquatic ones [40] as such, PNECs were calculated through conservative steps. PNECsoil was derived from PNECaquatic according to the simplified equilibrium partitioning (EqP) method [41,42].

(6) $$PNEC_{soil}=\frac{PNEC_{aquatic}\cdot K_{d}}{RHO_{soil}}$$

with Kd, the solid–water partition coefficient. RHOsoil the bulk density of wet soil (1.7 kg/L).

Kd values used are shown in Table 3.

**Table 3:** Kd values used for PNECsoil derivation and related references.

Substance	Koc Value	Kd Value in Soil (L/kg)	References
Carbamazepine		13	[43]
Ciprofloxacin		427	[44]
Diclofenac		9	[43]
Lincomycin	18.8–26.1	0.44	[45]
Ofloxacin		309	[46]
Pyrimethamine	590–1540	19.06	[47]
Sulfapyridine		8	[43]
Triclosan		127	[46]

With fOC = 0.02.

Experimentally derived Kd values were selected whenever possible. Two PhD theses also cited some Kd values from the bibliography. Whenever data were scarce, gray literature was sought, and Kd was calculated as Koc fOC. PNECaquatic values were found from NORMAN [48]. Due to very strong sorption [49] of QACs in soil, PNECsoil could not be calculated through the EqP method, and their risk was therefore not calculated.

For well-studied substances such as triclosan, PNECterrestrial was also calculated from toxicity endpoints for soil biota (plants and animals) following a tiered approach, USEPA ECOTOX KNOWLEDGE database [50] was consulted. If data were few or if they were not expressed in units/soil, additional reliable data from individual peer-reviewed studies were considered. NOECs and EC10s were preferred to EC50s, and the geometric mean of toxicity endpoints on different species was calculated. A Species Sensitivity Distribution calculation was performed on the ETX 2.0 software (RIVM, Bilthoven, The Netherlands), and the HC5 ratio was chosen with a safety factor of 5 to derive a PNECterrestrial.

It is noted that calculations were performed without considering further bioavailability of the soil pollutant towards soil-dwelling organisms, so as not to increase perplexity and uncertainty of calculations.

## 3. Results and Discussion

### 3.1. Sludge Production and Plant Characteristics

The ten investigated wastewater treatment plants (WWTPs) collectively treated approximately 167.3 Mm^3^ of wastewater per year, producing an estimated 70,431 t of dry sludge solids annually (Table 4). Considerable variation was observed among the plants in both treatment capacity and sludge yield, reflecting differences in population served, treatment technology, and operational conditions.

WWTP1-AS is the largest centralized treatment facility in Jordan, accounting for over three-quarters (76.7%) of the total national sludge production. In contrast, small to medium WWTPs such as MA, S, and WS each contributed less than 2% of the total sludge production, typically treating less than 5 Mm^3^/year of wastewater.

The sheer volume of nationally produced sludge annually (approximately 70,000 t) shows that large quantities of organic matter and nutrients can be produced and potentially be applied to Jordanian soils affected by desertification, low fertility, and increased salinity [51]. This application may positively affect crop production in the country [52,53], and it can also deter the unsustainable solution of landfilling [54].

It is noted once more that sludge hygienisation is imperative so that dangerous pathogens cannot reach humans through the food chain via crops or grazing animals [55]. As such, the present samples cannot be used as BS without further processing, and all data produced herein are applicable only after proper sanitization of the substrates. Previous research (2007) in Jordanian WWTPs utilizing drying beds showed that fecal coliform numbers prevented this sludge from being characterized as Class A (according to [18]) but it could well be classified as Class B. Subsequent analyses (2016–2019) from another WWTP showed high coliform numbers for the samples from the sludge thickener and lower but varied numbers for digested sludge [52].

### 3.2. Pollutant Load in Examined Sludge Samples

**Figure 2:**
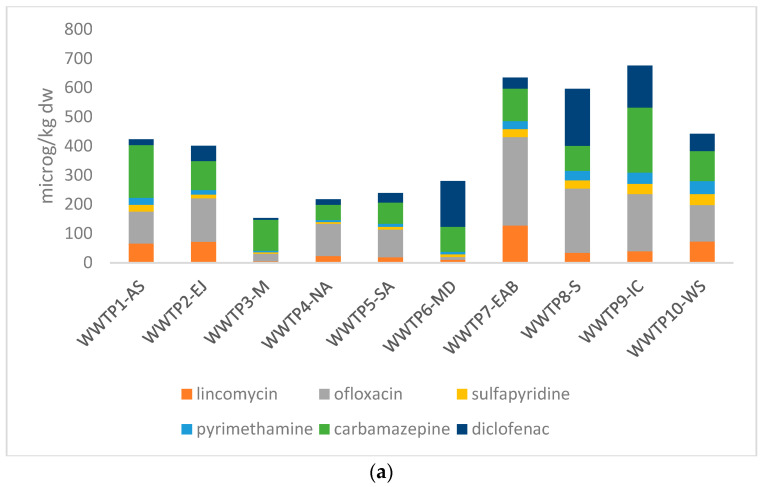

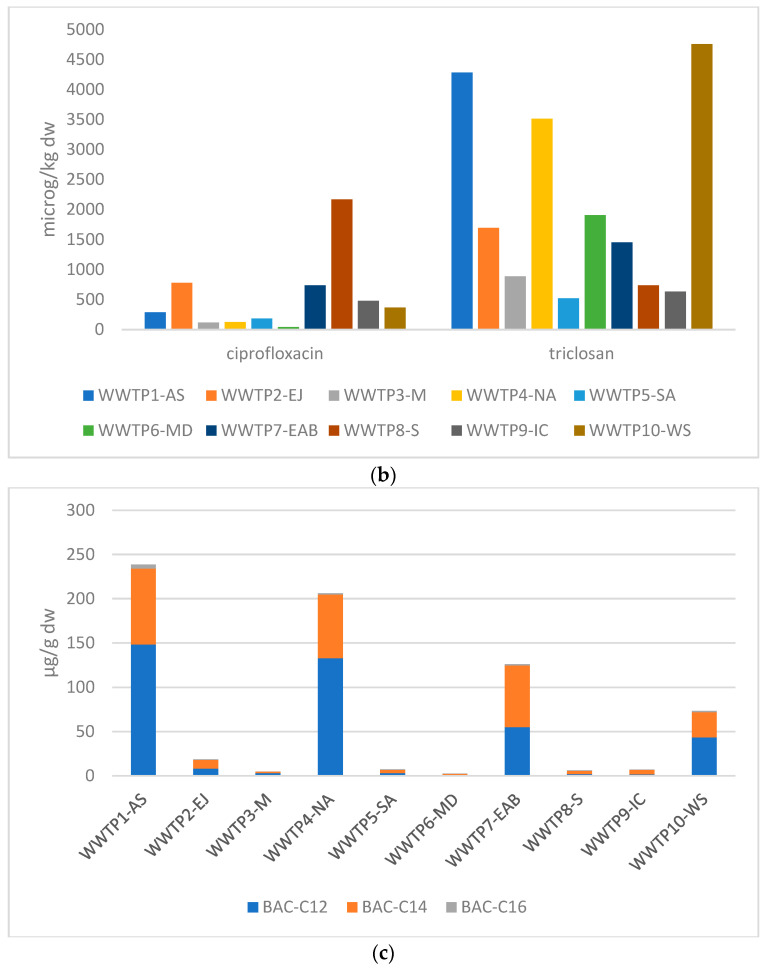
(**a**) Cumulative concentrations of 6 of the pollutants detected per WWTP; (**b**) comparison of ciprofloxacin and of triclosan concentrations per WWTP; (**c**) Cumulative concentrations of QACs (Benzyldodecyldimethylammonium chloride, Benzyltetradecyldimethylammonium chloride, Benzylhexadecyldimethylammonium chloride) detected per WWTP.

As in other cases of sludge of municipal origin [25], historical samples from WWTPs in Jordan did not show elevated concentrations of metals of toxicological concern [52,53]. Nevertheless, organic emerging pollutants are now rightfully considered as additional agents of concern in biosolids, and this is mirrored in national legislation of EU members and possibly in the future amendment of Directive 86/278/EEC [8]. For this reason, a total of eleven pharmaceutical compounds representing major antibiotic classes were analyzed in sludge samples collected from the examined WWTPs) across Jordan. The detected compounds included fluoroquinolones (ciprofloxacin, norfloxacin, ofloxacin, danofloxacin, flumequine, and nalidixic acid), lincosamide (lincomycin), sulfonamides (sulfadimidine, sulfamethoxazole, sulfapyridine), and trimethoprim. Other common drugs (one anticonvulsant, one NSAID) and several antimicrobial agents were also detected and quantified. Similar pollutants have been detected in sludge worldwide, as shown in Table 5. A visual representation of the present results is found in Figure 2.

Most antibiotics analyzed were detected at varying concentrations across the sampled WWTPs, ranging from below detection (<5 µg/kg) to 2165.6 µg/kg. The highest overall concentrations were observed in sludge from WWTP8-S (2165.6 µg/kg ciprofloxacin), WWTP7-EAB (302.9 µg/kg ofloxacin), and WWTP9-IC (480.2 µg/kg ciprofloxacin). In contrast, the lowest levels were recorded at WWTP6-MD (43.9 µg/kg ciprofloxacin) and WWTP3-M (<5 µg/kg for most antibiotics). These results indicate strong spatial variability. The present mean value of ciprofloxacin (329 µg/kg) is comparable to that found in Spain [56]. Very high to very low concentrations of this substance have been found in the USA (see Table 5). The predominance of fluoroquinolones agrees with global observations, where these compounds are known to adsorb strongly to sludge solids due to their cationic functional groups and high affinity for organic matter [57]. Their persistence in sludge reflects limited biodegradation during secondary and tertiary treatment. Lincomycin was also widely detected (10.3–127.4 µg/kg), with the highest concentration again found in WWTP7-EAB, followed by WWTP2-EJ and WWTP1-AS. Nalidixic acid was detected only in two sites (MD and EAB). Similar values of lincomycin have been found across the USA, but also around the world (see Table 5). These discrepancies for compounds such as fluoroquinolones, diclofenac, ibuprofen, sertraline, gemfibrozil, caffeine, and others between studies may be explained by the different prescription rates related to human health, sampling regions, or climate conditions. It has also been noted for large countries such as China that differences can be found within regions of the same country. Finally, for some countries, the representativeness of samples is low, with samplings from only one or a few WWTPs [58].

In the present samples, flumequine and danofloxacin occurred infrequently and at low concentrations, likely reflecting limited use. Sulfonamide antibiotics and trimethoprim were detected less frequently and typically at low concentrations. Among these, sulfapyridine was the most prevalent, detected in most sludge samples (6.1–37.6 µg/kg), while sulfamethoxazole and sulfadimidine appeared mainly in sludge from WWTP8-S and WWTP9-IC with maximum values of 47.4 µg/kg and 275 µg/kg, respectively. Higher but comparable concentrations of sulfapyridine have been found across the EU (see Table 3). Trimethoprim was only quantified in two WWTPs (22.1 µg/kg in WWTP7-EAB and 63.7 µg/kg in WWTP8-S), showing a similar distribution pattern to the sulfonamides. The extremely high concentrations of several antibiotics observed in WWTP8-S and WWTP9-IC may be attributed to partial influent from some pharmaceutical factories in the area. The presence of these industrial effluents introduces high-strength antibiotic residues into the municipal wastewater stream, leading to elevated levels in the final sludge. Industrial wastewater management enforcement is challenging in Jordan. Some factories operate on-site wastewater treatment plants to treat their industrial effluents, while others discharge their raw wastewater directly into municipal sewer networks and subsequently to domestic WWTPs. Moreover, pharmaceutical manufacturing facilities treat their wastewater to comply with the requirements of Jordanian Standard JS 202/2007 [59]; their treated effluents can contain elevated concentrations of substances not explicitly regulated under JS 202/2007. On the contrary, rural and less industrialized regions such as WWTP3-M and WWTP4-NA exhibited the lowest pharmaceutical levels, with many compounds below analytical detection limits.

Carbamazepine was detected in sludge samples from all ten investigated wastewater treatment plants (WWTPs), with concentrations ranging from 51.8 µg kg^−1^ (WWTP4-NA) to 223.4 µg kg^−1^ (WWTP9-IC). The compound showed relatively consistent distribution among the plants, confirming its ubiquitous presence and environmental relevance [60] (see also Table 5). Diclofenac is also a very environmentally relevant drug [60] (see also Table 5) and it was also detected in all sludge samples, with concentrations ranging from 6.0 µg kg^−1^ in WWTP3-M to 195.7 µg kg^−1^ in WWTP8-S. Intensive domestic consumption of diclofenac is noted, since this is one of the most widely used NSAIDs in Jordan for the treatment of pain, inflammation, and chronic diseases such as arthritis. WWTP3-M and WWTP4-NA exhibited markedly lower concentrations, consistent with their rural character, smaller resident population, and lower medical prescription rates.

Triclosan was detected in all sludge samples, with concentrations ranging from 521 µg kg^−1^ at WWTP5-SA to 4756 µg kg^−1^ at WWTP10-WS. The mean value noted here (1542 µg kg^−1^) is lower but comparable to other values found around the world (see Table 5). Triclosan is highly hydrophobic and exhibits a strong sorption affinity for organic matter, leading to its preferential accumulation in sewage sludge rather than remaining dissolved in the liquid effluent.

In parallel, quaternary ammonium compounds (QACs) are widely used in disinfectants, personal care products, fabric softeners, food-processing facilities, and healthcare institutions. Therefore, high concentrations of these compounds were detected in biosolids due to their strong cationic charge, which facilitates sorption onto negatively charged sludge particles, resulting in very low removal efficiencies through conventional treatment. QACs such as benzyldodecyldimethylammonium chloride (BAC-C12) and benzyldimethyltetradecylammonium chloride (BAC-C14) were the most abundant, with maximum concentrations of 148.4 µg g^−1^ and 85.8 µg g^−1^, respectively, both detected at WWTP-AS. Benzylhexadecyldimethylammonium chloride (BAC-C16) ranged from 0.13 to 4.62 µg g^−1^, whereas tetrabutylammonium bromide remained below the detection limit in all samples. QACs possess high sorption coefficients, leading to strong partitioning to sludge solids. Their persistence is enhanced by limited biodegradability under anaerobic or low-oxygen conditions [61], typical of thickening and dewatering processes. Overall, concentrations measured in this study (up to 148 µg g^−1^) are comparable with international reports, where total QACs in municipal sludge range between 22 and 343 µg g^−1^. The elevated levels in the large centralized WWTPs (AS, NA) reflect greater disinfectant usage and industrial contributions, while rural plants (M, MD) show minimal inputs consistent with lower cleaning-chemical consumption. Consequently, the reuse of sludge in agriculture containing QAC residues might be serving as a secondary reservoir for ARG dissemination into soil and crops.

The sampling locations across the ten wastewater treatment plants (WWTPs) represented different sludge processing stages, including dewatering outlets, drying beds, filter presses, thickeners, and secondary clarifiers. These operational differences play an important role in determining the final concentration of pharmaceuticals detected in the sludge. Overall, the variation in pharmaceutical concentrations among WWTPs reflects the combined effects of sludge treatment stage, local climate, and industrial or domestic inputs. Samples from mechanically thickened or dewatered sludge generally show higher accumulation of persistent compounds, while open drying beds may exhibit lower values due to natural attenuation processes such as sunlight exposure, volatilization, and microbial degradation. This pattern highlights the significant role of socio-economic and industrial factors in determining the occurrence and magnitude of pharmaceutical contamination in wastewater and sludge, a trend consistent with findings from other developing regions [62,63].

**Table 5:** Mean values of pollutants in sludge and some of their bibliographic values.

Substance	Present Median Value (μg kg^−1^)	Bibliographic Value (μg kg^−1^)	Country/Region	References
ciprofloxacin	329	2400–2700	Switzerland	[64]
		74.5–47,500	USA	[65]
		105–599	Spain	[56]
		11.33–145.42	Poland	[42]
		153.21	China	[10]
		60–12,858	Worldwide	[58]
		0.9–778	Worldwide	[66]
lincomycin	36	13.9–33.4	USA	[65]
		3.8	India	[58]
ofloxacin	117.5	73.9–58,000	USA	[65]
		1000	Sweden	[67]
		10–1000	Worldwide	[58]
		12.57–232.40	Poland	[42]
		2982.6	China	[10]
		0.1–510	EU	[66]
sulfapyridine	17.95	24–197	EU	[66]
carbamazepine	101.1	3.84–12,860	Worldwide	[58]
		69.6	Canada	[68]
		20.3–460	Spain	[41]
		4.7–89.7	Worldwide	[66]
diclofenac	44.85	4.1–330	EU	[58]
		10.4–424.7	Worldwide	[66]
triclosan	1542	2600–30,000	USA	[69]
		10–10,000	Sweden	[67]
		5580	Australia	[70]
		19100	Italy	[71]
		865–5940	Worldwide	[58]
		10–1508	Worldwide	[66]
various QACs	13.03 (sum) (µg/g)	not detected–6000 (µg/g)	Worldwide	[72]

Data for non-stabilized sludge unless otherwise stated.

### 3.3. Calculated Risk to Terrestrial Organisms

The present risk assessment was performed under the following caveats.

- The present sludge samples cannot be used as BS unless hygienization is verified.
- Risk assessment was performed for worst-case scenarios assuming no degradation of the pollutant at the time point of soil incorporation. However, whenever reliable data were available, TWA PECs instead of initial PECs were used as refinement.
- As stated in Section 2.4, toxicity data on terrestrial organisms are very scarce for most of the emerging pollutants. As such, the EqP method was used based on aquatic data, as also performed in [41]. For triclosan, PNEC was calculated through terrestrial data and compared to the value deriving from PNECaquatic via the EqP method, and to the PNECterrestrial calculated by [73].
- It was *a priori* assumed that each pollutant exerts its toxicity individually [74] as such, the total risk is the sum of all individual risks. In reality, synergy or antagonism may also be present; however, the concentration-addition approach as a Tier-1 screening option was adopted here [75]. It can be assumed that chemicals with similar actions (in this case the fluoroquinolones) can act in an independent-addition model. Furthermore, for pesticides and for metals, true synergistic effects have been rare in ecological models. Nevertheless, when different chemicals have effects on various taxonomic groups, this can lead to structural and functional changes in the ecosystem, which may supersede (be greater) than the added toxicities of the chemicals [75]. This is especially true for triclosan, a multi-stressor agent that showed a synergistic toxic effect with carbendazim on *D. magna* [76]. However, in other aquatic tests, chemical mixtures that include triclosan exerted either sub-additive or additive toxicity [77].

PEC values were calculated according to Formula (3). When a reliable DT50 in soil was available (carbamazepine, ciprofloxacin, lincomycin, diclofenac, triclosan), PEC values were calculated according to Formula (4).

Utilizing the proxy of aquatic data for the pollutants that were systematically above the LOQ, PNEC values were calculated for the substances shown in Table 2. A similar strategy was followed in [41,42]. Triclosan is a substance of particular concern because it is widely used and there are different opinions upon its safety [78]. Due to this heightened interest, several data were found in [50] database. However, after close inspection, out of 171 entries, the data on birds and mammals were excluded. Endpoints not expressed in kg or g of soil were also excluded. The remaining entries all corresponded to [79] and the actual study was retrieved and examined. Additional data was found in other peer-reviewed papers. All relevant references and the chosen NOECs are shown in Table 6.

It is possible that other short- or long-term studies for soil organisms and microorganisms or even plants are available for some other chemical substances if they are registered under the REACH Regulation in the EU, because there are legislatively binding requirements for their environmental risk assessment under some circumstances. Furthermore, veterinary medicinal products are required to undergo an environmental risk assessment as part of their authorization process under Regulation (EU) 2019/6. It is therefore possible that data that is not publicly available may substantially refine terrestrial toxicity assessment for some pollutants. It is important that whenever possible, these data become available to a wider audience for further research.

PNECterrestrial for triclosan was found as described in Section 2.4 and it was equal to 0.086 μg triclosan/kg soil.

**Figure 3:**
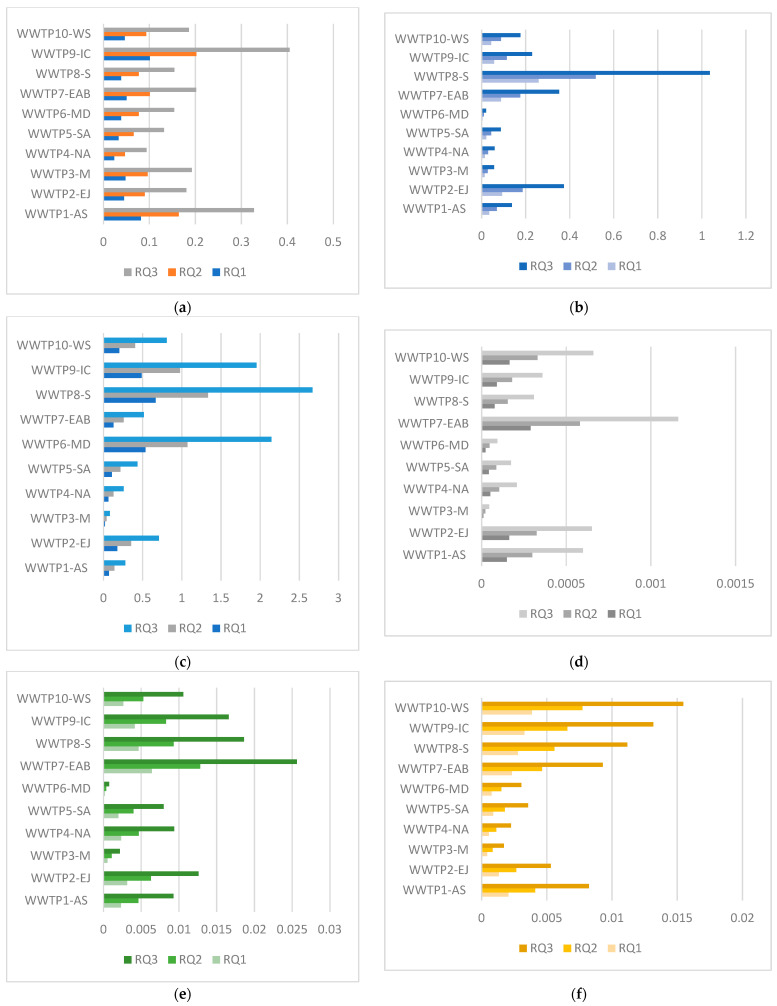

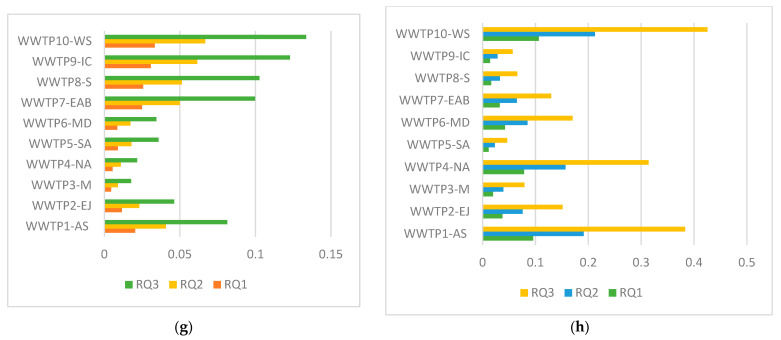
Risk quotients for the 8 pollutants detected in sludge. RQ above 1 is not acceptable (**a**) carbamazepine, (**b**) ciprofloxacin, (**c**) diclofenac, (**d**) lincomycin, (**e**) ofloxacin, (**f**) pyrimethamine, (**g**) sulfapyridine, (**h**) triclosan. RQ1 is the calculated risk for a dose of 5 t BS/ha, RQ2 is for 10 t/ha, and RQ3 is for 20 t/ha.

The risk quotient PEC/PNEC for each substance is shown in Figure 3.

As deduced from Figure 3, risk is acceptable for substances with higher PNECaquatic (above 1 μg/L), such as pyrimethamine (PNECaquatic = 2 μg/L), which exhibits a modest calculated kd (19.06 L/kg). Carbamazepine (PNECaquatic = 0.5 μg/L) and sulfapyridine (PNECaquatic = 0.46 μg/L) also do not pose a significant risk since PEC values were quite modest even at the putative scenario of 20 t/ha. Ofloxacin (PNECaquatic = 0.5 μg/L) is also much less mobile (kd = 309 L/kg) and did not pose a significant risk.

Triclosan shows a PNECaquatic = 1.04 μg/L and a kd = 127 L/kg); however, it should be noted that the PNECterrestrial used for Figure 3 was derived from terrestrial data, and it was equal to 0.086 μg/kg. The PNECterrestrial calculated via the EqP method was very similar and equal to 0.077 μg/kg. An interesting study by [73] calculated the PNECterrestrial using real data from toxicity assays on terrestrial organisms (chronic exposure; earthworms, and five plant species). The proposed PNECs varied from 0.04 to 0.021 μg/kg when a log-logistic SSD was used and from 0.09 to 0.44 μg/kg when a log-normal SSD was used. The lowest values were calculated utilizing a safety factor of five, as also performed here, and the value 0.09 μg/kg is very close to the PNECterrestrial proposed here. Nevertheless, triclosan is a ubiquitous chemical that already demonstrates a significant library of ecotoxicity studies; this is not the case for other emerging chemicals, especially for soil-based ecosystems.

The remaining pollutants, ciprofloxacin and especially diclofenac, produced RQs above the value of 1 for WWTP6-MD, WWTP7-EAB, and WWTP8-S for some possible scenarios of fertilization (5–20 t/ha).

The cumulative risk is shown in Table 7 for all 10 WWTPs, and it is assumed that the risk is additive.

As can be seen from the data, WWTP3-M, WWTP4-NA, and WWTP5-SA consistently gave sludge of low environmental risk. These WWTPs are situated in rural areas with a smaller resident population, as stated before. On the contrary, WTTP8-S and WWTP9-IC showed high risk, which may be attributed to partial influents from some pharmaceutical factories in the area. The sludge from these areas should therefore not be used as fertilizers. It is not advisable to continue the landfilling of this waste either; it should be considered whether a nation-wide investment in an incineration or co-incineration facility is feasible [89]. The maintenance costs could be partially covered through heat production, while the produced ash can be incorporated in cement. In any case, it is imperative that new technologies, such as tertiary treatment, are applied to the pharmaceutical industries of the examined areas so that their effluents are less encumbered when leaving the plant. It is also interesting to note that sludge, which was partially treated in drying beds, was of low risk in the cases of WWTP3-M and WWTP-5, but the risk was high for WWTP6-MD, which also uses this process. It is not always possible to sequester pollutants during sludge processing; for example, sludge-derived biochar showed varied toxicity to earthworms relative to the temperature at which the biochar was produced [90]. Furthermore, additional treatment of biosolids from a WWTP in Northern Greece caused a small increase in leaching of Ni and Zn [25]. Data on the largest WWTP (AS), which furnishes a large city and produces high amounts of sludge, showed that this waste can indeed become a good substrate for BS, if properly treated, sanitized, and used at low doses and/or at low frequency for the same soil. In any case, it is very important to periodically assess the ecotoxicity of these sludges before their application [8,91].

The limitations of the present research have been mentioned in other parts of the present paper (reproducibility and representativeness of samples, restrictions in chemical analysis, and in available databases). As such, the present results can be characterized as preliminary, and recommendations for future work can be summarized as follows:

- The sludges deemed as suitable should be properly sanitized and checked for heavy metal content and HACs such as PCBs and PAHs, before applied to land.
- More sludge sampling campaigns are necessary to ensure repeatability and reproducibility of results and to enable spatial statistical comparisons for the WWTPs.
- Information on the fate and ecotoxicity of pharmaceuticals in sludge-amended soils should be actively sought and made publicly available, particularly if such data are included in regulatory registration dossiers submitted to competent authorities.
- The additive toxicity model can be used as a Tier I evaluation; however, reliable data on possible antagonism or synergy should also be sought.

## 4. Conclusions

The present research investigated the pollutant load in sewage sludge samples from 10 WWTPs in Jordan. Some of the samples were not treated, such as those taken from the secondary clarifier, while others were minimally treated, such as those that were thickened, pressed, or dried. The WWTPs included ones from large urban agglomerations, from rural areas, while some of them also received inlets from industry. The pollutants that consistently gave values above the limit of quantitation in an LC–MS/MS system were carbamazepine, ciprofloxacin, diclofenac, lincomycin, ofloxacin, pyrimethamine, sulfapyridine, and triclosan. QACs were also detected via HPLC-MS/MS, and BAC-C12, BAC-C14, and BAC-C16 were found in all WWTPs examined. The level of pollution in sludge samples was comparable to findings worldwide, at the lower end of the data. Risk to soil-dwelling organisms (flora and fauna) was calculated for all eight chemicals from aquatic data based on the EqP method for three putative levels of fertilizing use (5, 10, and 20 t/ha), while for QACs, this method cannot be reliably applied. Especially for triclosan, risk was also calculated using data on terrestrial (non-mammalian and non-avian) organisms. Results show that the risk is mainly driven by the NSAID diclofenac and that it was non-acceptable, particularly for 3 out of the 10 WWTPs. On the contrary, sludges from three rural areas were of lower risk and they could possibly be applied to nearby agricultural land if they are stabilized and sanitized. This is the first study to assess the environmental impact of sludge application in Jordan and it also highlights the data gaps still present in terrestrial risk assessment for many circular economy initiatives.

## Figures and Tables

**Table 1:** Main characteristics of the examined WWTP in Jordan.

WWTP Site	WW Flow (Mm^3^/y)	Sampling Point	TSS (mg/L) (Influent)
WWTP1-AS	125.8	Dewatering outlet	429
WWTP2-EJ	1.8	Dewatering outlet	772
WWTP3-M	2.1	Drying beds	321
WWTP4-NA	8.8	Filter press outlet	268
WWTP5-SA	8.4	Drying beds	323
WWTP6-MD	2.9	Drying beds	958
WWTP7-EAB	5.8	Thickener outlet	457
WWTP8-S	3.8	Secondary Clarifier outlet	309
WWTP9-IC	3.2	Thickener outlet	430
WWTP10-WS	4.7	Dewatering outlet	285

WW flow: influent rate to the WWTP per year. TSS: total suspended solids in the influent.

**Table 4:** Produced annual quantities of sludge from the examined WWTPs.

WWTP Site	Sludge Production t/y (Dry Solid)	Percent %
WWTP1-AS	54,001	76.7
WWTP2-EJ	1390	2.0
WWTP3-M	684	1.0
WWTP4-NA	2343	3.3
WWTP5-SA	2703	3.8
WWTP6-MD	2797	4.0
WWTP7-EAB	2639	3.7
WWTP8-S	1162	1.6
WWTP9-IC	1387	2.0
WWTP10-WS	1325	1.9

**Table 6:** Reliable toxicological endpoints for soil biota for triclosan.

Substance	Organism	Endpoint Type NOEC	Endpoint Value	References
triclosan	juvenile *Eisenia fetida*	weight gain	100 μmol/kg (28.9 mg/kg)	[79]
	*Eisenia fetida*	weight gain	10 mg/kg (lowest of 4 exp. conditions)	
	*Eisenia fetida*	growth rate	10 mg/kg	
	*Eisenia fetida*	number of juveniles	10 mg/kg	[80]
	*A. vulgare*	alkaline phosphatase enzyme activity	1.35 mg/kg	[81]
	*L. sativa*	seedling length	1.35 mg/kg	
	*S. alba*	seedling length	1.35 mg/kg	
	*E. fetida*	lethality	1 mg/kg	[82]
	Microbiota	respiration	10 mg/kg soil	[83]
	Microbiota	respiration	2 mg/kg soil	
	*Cucurbita pepo*	plant biomass increase	1 mg/kg soil	
	*E. fetida*	DNA damage	1 mg/kg soil	[84]
	*E. fetida*	growth inhibition rate	10 mg/kg soil	[85]
	*E. andrei*	juvenile weight	<35 mg/kg soil *	[86]
	microbiota	N cycle efficacy	5 mg/kg soil	[87]
	microbiota	respiration	10 mg/kg soil	
	*Achatina fulica*	biomass, shell diameter growth, and total food intake	24 mg/kg soil	[88]

* Only concentration tested.

**Table 7:** Cumulative risk to soil organisms for 5, 10, and 20 t BS/ha.

WWTP Site	RQ1 (Total)	RQ2 (Total)	RQ3 (Total)
WWTP1-AS	0.31	0.61	**1.23**
WWTP2-EJ	0.37	0.74	**1.48**
WWTP3-M	0.11	0.22	0.44
WWTP4-NA	0.19	0.38	0.76
WWTP5-SA	0.19	0.37	0.75
WWTP6-MD	0.63	**1.26**	**2.53**
WWTP7-EAB	0.33	0.67	**1.34**
WWTP8-S	**1.01**	**2.03**	**4.06**
WWTP9-IC	0.70	**1.4**	**2.80**
WWTP10-WS	0.44	0.88	**1.76**

Values in bold are above the risk threshold of 1.

## Data Availability

The raw data supporting the conclusions of this article will be made available by the authors on request.

## Supplementary Materials

The following supporting information can be downloaded at: https://www.mdpi.com/article/10.3390/toxics14010062/s1, Table S1: Limit of Quantification (LOQ) for Target Pharmaceutical Compounds in Soil Samples (LC–MS/MS). Table S2: Limit of Quantification (LOQ) for Target Pharmaceutical Compounds in Water Samples (LC–MS/MS).

## Abbreviations

The following abbreviations are used in this manuscript:

BAC-C12	Benzyldodecyldimethylammonium chloride
BAC-C14	Benzyltetradecyldimethylammonium chloride
BAC-C16	Benzylhexadecyldimethylammonium chloride
HACs	Halogenated aromatic compounds
QACs	Quaternary ammonium compounds
PEC	Predicted environmental concentration
PNEC	Predicted no effect environmental concentration
RQ	Risk quotient
SSD	Species sensitivity distribution
WWTP	Wastewater treatment plant
