# Supplementary material for: Quantification of Pharmaceuticals in Sludge Produced from Wastewater Treatment Plants in Jordan and Environmental Risk Assessment
Source: Toxics. 2026 Jan 8;14(1):62. doi: 10.3390/toxics14010062 (PMC12846146; doi:10.3390/toxics14010062)
Supplement: Supplementary file 1 [file toxics-14-00062-s001.zip › toxics-4074553-supplementary.pdf]

Table S1: Limit of Quantification (LOQ) for Target Pharmaceutical Compounds in Soil Samples (LC–MS/MS)

**Compound**	**LOQ (µg/kg)**
Ampicillin	0.5
Carbamazepine	0.2
Cefazolin	0.5
Ceftiofur	0.5
Cephapirin	0.5
Chloramphenicol	0.5
Ciprofloxacin	0.2
Cloxacillin	0.5
Danofloxacin	0.5
Diaveridine	0.5
Diclofenac	0.5
Dicloxacillin	0.3
Difloxacin	0.5
Enrofloxacin	0.5
Erythromycin	0.5
Flumequine	0.5
Lincomycin	0.5
Marbofloxacin	0.5
Miloxacin	0.5
Nalidixic Acid	0.5
Norfloxacin	0.5
Ofloxacin	0.5
Orbifloxacin	0.5
Ormetoprim	0.5
Oxolinic Acid	0.5
Oxytetracycline	0.5
Pimodic Acid	0.4
Progesterone (Hormone)	0.1
Pyremethamine	0.5
Pyrimethamine	0.5
Sarafloxacin	0.5
Sulfabenzamide	0.5
Sulfacetamide	0.5
Sulfadiazine	0.5
Sulfadimethoxine	0.5
Sulfadimidine	0.4
Sulfadoxine	0.5
Sulfaethoxypyridazine	0.5
Sulfaguanidine	0.5
Sulfamerazine	0.5
Sulfamethoxazole	0.5
Sulfamethoxypyridazine	0.5
Sulfamonomethoxine	0.5
Sulfanitran	0.5

Sulfapyridazine	0.5
Sulfapyridine	0.5
Sulfaquinoxaline	0.5
Sulfathiazole	0.5
Sulfatroxazole	0.5
Sulfisomidine	0.5
Sulfisoxazole	0.5
Testosterone (Hormone)	0.1
Tetracycline	0.5
Thiamphenicol	0.5
Triclosan	0.5
Trimethoprim	0.5

Table S2: Limit of Quantification (LOQ) for Target Pharmaceutical Compounds in Water Samples (LC–MS/MS)

**Compound**	**LOQ (ng/L)**
Ampicillin	1
Carbamazepine	0.4
Cefazolin	1
Ceftiofur	1
Cephapirin	1
Chloramphenicol	1
Ciprofloxacin	0.4
Cloxacillin	1
Danofloxacin	1
Diaveridine	1
Diclofenac	1
Dicloxacillin	0.6
Difloxacin	1
Enrofloxacin	1
Erythromycin	1
Flumequine	1
Lincomycin	1
Marbofloxacin	1
Miloxacin	1
Nalidixic Acid	1
Norfloxacin	1
Ofloxacin	1
Orbifloxacin	1
Ormetoprim	1
Oxolinic Acid	1
Oxytetracycline	1
Piromidic Acid	0.8
Progesterone (Hormone)	0.2
Pyremethamine	1
Pyrimethamine	1
Sarafloxacin	1
Sulfabenzamide	1
Sulfacetamide	1
Sulfadiazine	1
Sulfadimethoxine	1
Sulfadimidine	0.8
Sulfadoxine	1
Sulfaethoxypyridazine	1
Sulfaguanidine	1
Sulfamerazine	1
Sulfamethoxazole	1
Sulfamethoxypyridazine	1
Sulfamonomethoxine	1
Sulfanitran	1

Sulfapyridazine	1
Sulfapyridine	1
Sulfaquinoxaline	1
Sulfathiazole	1
Sulfatroxazole	1
Sulfisomidine	1
Sulfisoxazole	1
Testosterone (Hormone)	0.2
Tetracycline	1
Thiamphenicol	1
Triclosan	1
Trimethoprim	1
